# Band Gap and Reorganization Energy Prediction of Conducting Polymers by the Integration of Machine Learning and Density Functional Theory

**DOI:** 10.1021/acs.jcim.5c00345

**Published:** 2025-05-28

**Authors:** Tugba Haciefendioglu, Erol Yildirim

**Affiliations:** α Department of Chemistry, 52984 Middle East Technical University, 06800 Ankara, Turkey; β Department of Polymer Science and Technology, Middle East Technical University, 06800 Ankara, Turkey; γ Department of Micro- and Nanotechnology, Middle East Technical University, 06800 Ankara, Turkey

## Abstract

The performance and reliability of machine learning (ML)-quantitative structure–property relationship (QSPR) models depend on the quality, size, and diversity of the data set used for model training. In this study, we manually curated a large-scale data set containing 3120 donor–acceptor (D–A) conjugated polymers (CPs) by selecting the most utilized 60 donors and 52 acceptors. This data set serves as a valuable resource for ML-based prediction of key electronic properties such as band gap energy (*E*_g_) and hole reorganization energy (λ_h_), calculated using density functional theory (DFT) to advance organic photovoltaics (OPV). Beyond data set construction, we systematically investigated how different descriptor and fingerprint types impact performance of the ML model. Recognizing that not all features contributed equally to the model performance, we conducted an in-depth analysis to identify the most informative descriptors for the fundamental optoelectronic properties. Our findings show that kernel partial least-squares (KPLS) regression utilizing radial and molprint2D fingerprints achieved the highest accuracy in predicting *E*_g_, with *R* ^2^ values of 0.899 and 0.897, respectively. For λ_h_ prediction, models integrating electronic descriptors such as frontier orbital energy levels significantly improved performance, achieving an *R* ^2^ value of 0.830. This study provides a comprehensive investigation of how different descriptors influence model performance in OPV research. By analyzing why certain models succeed while others fail, our findings offer insight into feature selection and data set optimization for accurate target property prediction in organic electronics. The developed ML models provide a predictive framework for high-performance OPV materials design, significantly reducing the reliance on labor-intensive experimental procedures and computationally expensive first-principle calculations.

## Graphical abstract

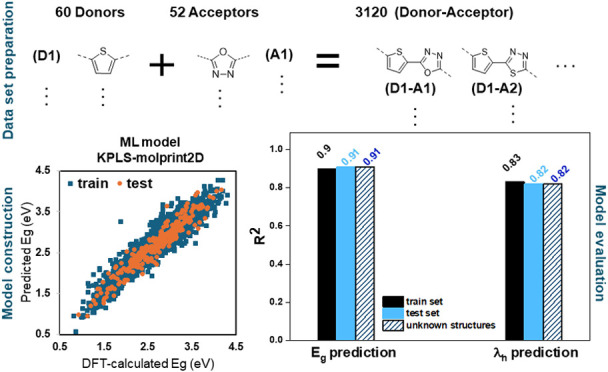

## Introduction

Recently, the power conversion efficiency (PCE) of organic photovoltaics (OPV), particularly those employing bulk heterojunction (BHJ) architecture, has surpassed 20%.^1^ The BHJ morphology consists of a blend of donor (D) and acceptor (A) moieties, which facilitates the generation of renewable solar energy. Characteristics such as lightweight, low cost, ease of processing, and flexibility render these conjugated polymers (CPs) effective and popular candidates for OPV applications compared with traditional solar cells.^2–4^

Donor units play a major role in determining the highest occupied molecular orbital (HOMO) while acceptor units exert a stronger influence on the lowest unoccupied molecular orbital (LUMO) energy level.^5^ This understanding is fundamental in the rational design of D–A CPs which typically involves a combination of an electron donor with a strong electron-withdrawing unit within a polymer chain.^6^ D–A copolymers, where both units are covalently linked in a single chain, are widely utilized in OPV systems.^7,8^ This design improves charge transfer and allows fine-tuning of HOMO–LUMO energy levels, aiming to achieve higher open-circuit voltage (*V*_oc_) and short-circuit current density (*J*_sc_), both critical parameters for enhancing PCE. However, optimizing PCE requires a careful balance between the gains and losses in *V*_oc_ and *J*_sc_ that require screening hundreds of combinations of donor and acceptor moieties. In addition to *V*_oc_ and *J*_sc_, several electronic properties such as energy levels, charge carrier mobility, and optical absorption characteristics are crucial in influencing the performance of the solar cells.^5,9^ These properties need careful optimization to ensure high efficiency.

Among the key factors influencing OPV efficiency, the band gap (*E*_g_), which is defined as the energy difference between the HOMO and LUMO energy levels, is particularly critical in the engineering of OPVs.^10^ Despite the availability of computational techniques, obtaining and screening the *E*_g_ of donor–acceptor CPs in a convenient and efficient manner remains a significant challenge.^11–13^ Although an approximation would suggest *E*_g_ ≈ LUMO­(A)–HOMO­(D), orbital hybridization and conjugation effects in real D–A structures cause significant deviations from this simple model. Therefore, a full quantum mechanical evaluation of the combined system is necessary to capture these perturbative effects, which cannot be predicted from the isolated fragment properties alone. Furthermore, the band gap should extend into long wavelength regions such as red and even infrared regions of the solar spectrum to obtain higher flux, resulting in a smaller energy gap of around 1.1–1.4 eV.^14^ This broadening of the solar spectrum enhances light-harvesting efficiency; however, it may lead to a reduction in *V*_oc_, thereby decreasing the overall PCE for the lower values of *E*_g_. In addition, charge carrier mobility is another parameter showing the movement of the electrons and holes under an electrical field.^15^ Higher mobility leads to more efficient charge transport, which is essential for maximizing the *J*_sc_ in solar cell applications. According to the Marcus theory of electron transfer, the charge transfer rate is influenced by charge reorganization energy which reflects the geometric and electronic changes occurring during the charge transfer process.^16^ A lower reorganization energy is one of the most important factors for higher charge mobility. Therefore, positive and negative effects that enhance and reduce efficiency should be thoroughly evaluated, and the optimal parameters should be determined to aim the highest efficiency for organic electronic applications.

The traditional approach to characterizing and screening these electronic and photovoltaic properties involves several steps, starting with material design, followed by chemical synthesis and extensive purification processes.^17^ The next step is the material characterization which is categorized as chromatographic, rheometric, thermal, spectroscopic, or microscopic that can be laborious and expensive due to practically inefficient trial-and-error examinations.^18^ This time-consuming cycle can take hours to days, and even after completion, the desired molecule with the target properties may not be obtained. Alternatively, computational methods, such as density functional theory (DFT), can provide high-accuracy predictions for the characterization of materials.^19–22^ While DFT offers a faster approach to understanding the properties of donor–acceptor CPs, it still requires substantial computational resources and advanced computing platforms.^23^ Therefore, a new approach to materials discovery is crucial for identifying effective donor–acceptor pairs in next-generation OPV applications. Here, we propose a machine learning (ML) approach which offers a cost-effective and fast alternative allowing the prediction of characteristics of CPs such as electronic *E*_g_, and hole and electron reorganization energies (λ_h_ and λ_e_). The determination of *E*_g_ through ML model trained on structural descriptors significantly advances material design.^11,24–27^ In addition, the reorganization energy represents the total energy required for a neutral molecule to relax from the ionic geometry to its optimized neutral geometry and vice versa. It is a critical parameter for CPs, as it directly impacts charge carrier mobility in organic electronic applications.^28^

Quantitative structure–activity relationship (QSAR) and quantitative structure–property relationship (QSPR) analyses develop an algorithm that estimates and predicts physical or chemical characteristics of a new compound, knowing only the values of that property for pre-studied compounds.^29^ QSPR method is an ML method that uses 2D or 3D structural information (descriptors) which determines the success of target property prediction.^30^ However, material descriptors including constitutional, topological, geometric, atomic, and molecular properties, as well as polymer fingerprints, vary widely.^25^ Identifying the most relevant descriptors for accurate property prediction is crucial for experimental design in the next step. Each type of descriptor may not cover all properties of materials; for instance, polymer fingerprints account only for the composition of the polymer, ignoring the other structural details.

While many prior studies have applied machine learning to predict the electronic properties of organic materials, our aim in this work is not to propose a new ML method but to deliver a property-specific benchmarking of diverse descriptor families on a manually curated and physically realistic data set. By exploring how descriptor types perform across structurally similar and dissimilar molecules, we provide critical insight into the model generalizability and the suitability of different feature sets for predicting distinct electronic properties. In this study, a combination of DFT computations and ML algorithms, including linear and nonlinear regression methods, was utilized to predict *E*_g_ and λ values using the largest CP database in the literature. Initially, the results of the DFT method were employed to calculate *E*_g_ and λ values of the training set for ML. Then, ML models were constructed to predict these target parameters based on the descriptors generated using DFT calculations. Therefore, ML models enable the rapid prediction of *E*_g_ and λ values of all D–A-type CPs, eliminating the need for labor-intensive experiments or high-cost DFT calculations.

## Computational Methods

### Data Set Preparation

The first step involved collecting data on D–A-type CPs from the literature. A total of 52 commonly used acceptor units from our previous study and 60 donor units were selected, as illustrated in Figures S1 and S2.^19,22^ These structures are successfully synthesized and characterized, focusing on D and A units commonly studied in organic electronic applications. The point where D and A units are connected to each other and the construction of the data set containing D–A monomers are demonstrated in Figure S3. Each D and A unit was included only once in the data set, regardless of the presence of two attachment sites. In cases where the D or A core was asymmetric, only one possible D–A combination was considered to maintain consistency. To generate the data set, 3120 D–A structures were constructed through R-group enumeration using a genetic algorithm in the Schrödinger, Materials Science Suite, ensuring a 1:1 donor-to-acceptor formation ratio.^31^ Several preprocessing steps such as cleaning and validating the structures were performed, ensuring structural accuracy before further optimization calculations.

### Molecular Descriptor Extraction

The band gap of the data set was calculated using optimization calculation with B3LYP functional and 6–311+G­(d) basis set in Jaguar Software.^32,33^ These DFT-calculated band gap values served as a reference for evaluating the accuracy of ML-based predictions. Similarly, hole (λ_h_) and electron (λ_e_) reorganization energies were determined using an optoelectronic calculation algorithm using the same software.^32^ These parameters were selected as the target properties for the ML model construction.

Unlike popular open-source descriptors such as Morgan, Mordred, and GNN-based representations, we employed feature sets natively supported by the Schrödinger AutoQSAR platform. These include various cheminformatic descriptors and 2D fingerprints, which have been optimized and extracted for regression performance within this environment, including polymer characteristics, properties calculated by semiempirical methods (PM3), cheminformatic descriptors, functional group counts, and atomic and molecular descriptors. These descriptors were generated for the entire data set. Additionally, the automated QSAR (AutoQSAR) was used to compute four different types of binary fingerprints (dendritic, linear, radial, and MOLPRINT2D) retaining the 10,000 most informative bits for each fingerprint type and several hundred numerical 2D descriptors (molecular, topological, and feature counts). Periodicity was explicitly considered during the generation of polymer-specific descriptors and polymer fingerprints by constructing representative oligomeric fragments that reflect the repeating nature of donor–acceptor polymer chains. These fragments were used to encode structural features, such as backbone connectivity and extended conjugation. In contrast, cheminformatic descriptors and general binary molecular fingerprints were computed from monomeric D–A structures since they focus on local molecular environments and do not require periodic representation. A list of descriptors is given as Supporting Information. Since many of these descriptors exhibit strong correlations, a feature selection process was applied to identify the most informative subset, ensuring that all correlation coefficients remained below a defined specified threshold. Descriptors with identical values in more than 90% of the data set were excluded since they provided minimal variability for property prediction.

### Model Construction

The QSAR models were built using AutoQSAR, an automated ML tool integrated into Maestro, Schrödinger, following previously established methodologies.^34,35^ The data set was randomly split into 80% training and 20% test set for model development and validation. To construct predictive models, various regression techniques were applied, including kernel-based partial least-squares regression (KPLS) and multiple linear regression (MLR) were adopted to build the set of models.^36^ Initially, the training and test sets were automatically determined, and at least 100 ML models were built, enforcing a maximum allowed correlation of 0.99 between any pair of independent variables. The best-performing model was then determined based on the predefined evaluation criteria and was used to predict the target properties of 10 unseen molecules (unseen by the algorithm). Finally, model prediction was assessed using prediction evaluation criteria to determine their reliability.

### Validation and Evaluation

The best ML model was selected based on internal and external validation parameters, ensuring both robustness and generalizability. The evaluation criteria included internal validation of the regression coefficient (*R* ^2^) and the standard deviation (SD) for the training set and external validation parameters such as the predicted regression coefficient (*Q* ^2^) and the root-mean-square error (RMSE) for the test set compounds. To improve prediction accuracy, a consensus model was also implemented combining predictions from multiple top-performing models. The performance of individual and consensus models was assessed using key statistical metrics including *R* ^2^, SD, RMSE, and mean average deviation (MAD). These evaluation parameters provided a comprehensive assessment of the accuracy and reliability of the ML models, ensuring their applicability for predicting the *E*_g_ and λ_h_ values in donor–acceptor CPs.

## Results and Discussion

For ML-based QSPR/QSAR applications, data set plays a crucial role in determining the performance and reliability of models. The size and diversity of the data set significantly impact the generalizability and accuracy of the constructed ML model. A sufficiently large data set ensures adequate training without underfitting, which occurs when the model fails to capture underlying trends. In this study, data sets were constructed by selecting the most used D and A units from organic electronic applications of these CPs. A key feature of this study is the construction of a manually curated data set containing 3120 unique D–A CPs. These structures are assembled from experimentally reported donor and acceptor units and optimized using B3LYP/6–311+G­(d) level DFT calculations. To our knowledge, this constitutes one of the largest and most chemically relevant DFT-optimized data sets available for organic polymer electronics and it is made publicly available to the research community (see the Supporting Information). The distribution of *E*_g_, λ_h_, and λ_e_ is determined by DFT calculations for all 3120 structures demonstrated in Figure 1. This distribution highlights the diversity of the data set for *E*_g_, enabling ML models to learn and generalize across a wide range of chemical structures and properties, thereby enhancing the applicability for predicting new materials. Since the distribution is wider than λ_e_ and its stronger relevance to hole transport in OPVs, ML-based prediction of only λ_h_ was performed using DFT-calculated values.

**Figure 1:**
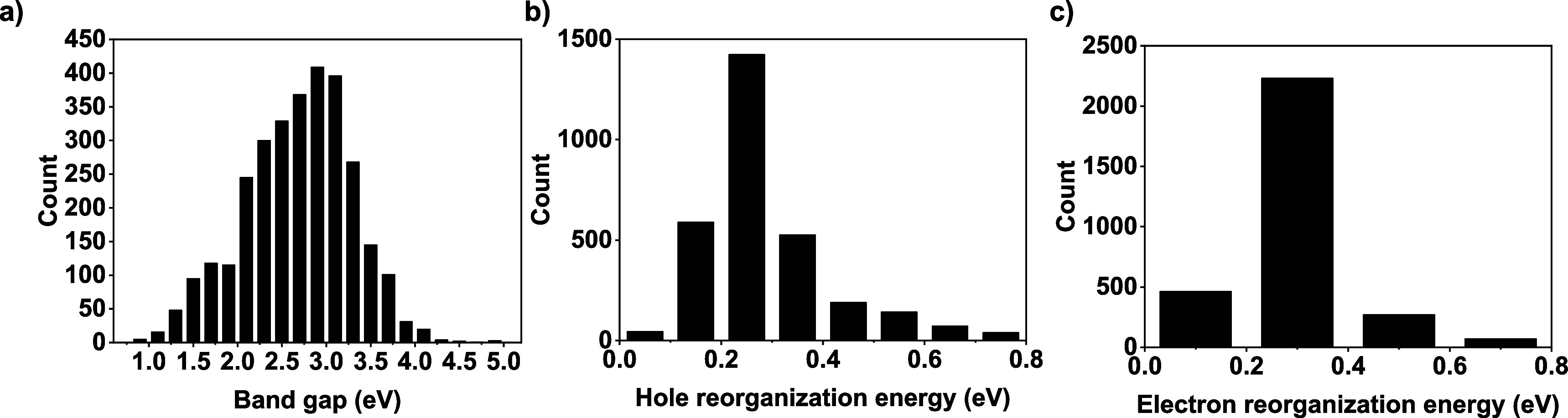
Histogram showing the distribution of (a) band gap, (b) hole reorganization energy, and (c) electron reorganization energy of the data set. Count shows the number of D–A molecules.

The chemical composition of materials defines their functionality and suitability for various applications. The properties of organic materials can be represented using molecular descriptors, which can be computed quickly and efficiently, or using quantum chemical descriptors, which require more computational effort. Molecular descriptors provide a numerical representation of molecular characteristics and their collection serves as a valuable tool for predicting a wide range of properties. However, the importance of each component is crucial, as some descriptors play a more significant role in prediction than others.

### QSPR on Band Gap Prediction

The type of ML models and descriptors used significantly affect the performance of machine analysis. In this study, different descriptor types were employed to predict the band gap of the data set using the QSPR method. Among the DFT-calculated nonzero band gaps in the database we created for this study, 80% (2496 D-A) were randomly selected as a training set, while 20% (624 D-A) were used as the test set to assess the accuracy of ML models in predicting of unknown molecules. The ML routine was utilized for the random allocation of molecules into training and test sets. The statistical performance of QSPR models based on different types of descriptors and fingerprints is summarized in Table 1 and scatter plots for ML models are shown in Figure 3. Additionally, identities and feature selection details for these ML models are given in Table S1.

**Table 1:** Performance of ML Models in Band Gap Prediction

**ML model**	**model score**	**RMSE**_**train**_	***R*** ^**2**^	**RMSE**_**test**_	***Q*** ^**2**^
**kpls_desc_33**	0.897	0.193	0.898	0.194	0.898
**kpls_desc_11**	0.115	0.506	0.073	0.493	0.118
**mlr_53**	0.493	0.385	0.465	0.363	0.522
**mlr_72**	0.562	0.356	0.542	0.337	0.589
**mlr_31**	0.686	0.290	0.686	0.600	0.686
**kpls_radial_35**	0.899	0.192	0.899	0.190	0.900
**kpls_molprint2D_11**	0.897	0.200	0.895	0.177	0.914
**kpls_dendritic_26**	0.826	0.192	0.855	0.227	0.839
**kpls_linear_81**	0.719	0.173	0.891	0.237	0.796

Numerical descriptors of the data set including molecular, topological indices, and physiochemical properties excluding the molecular orbital energy values were used for band gap prediction, resulted in the best performance **kpls_desc_33** which achieved an *R* ^2^ of 0.898, a low RMSE of 0.194 for the training set, and *Q* ^2^ of 0.898 for test set as shown in Table 1 and Figure 2. High predictive performance of this KPLS regression-based model shows that these numerical properties such as graph-based connectivity indices, electronegativity, and dipole moment are associated with band gap prediction as they influence electron delocalization and band structure. Rapid and computationally inexpensive calculation of molecular descriptors enables their use in molecular engineering for D–A CPs, thus facilitating the design of materials with tailored electronic properties.

**Figure 2:**
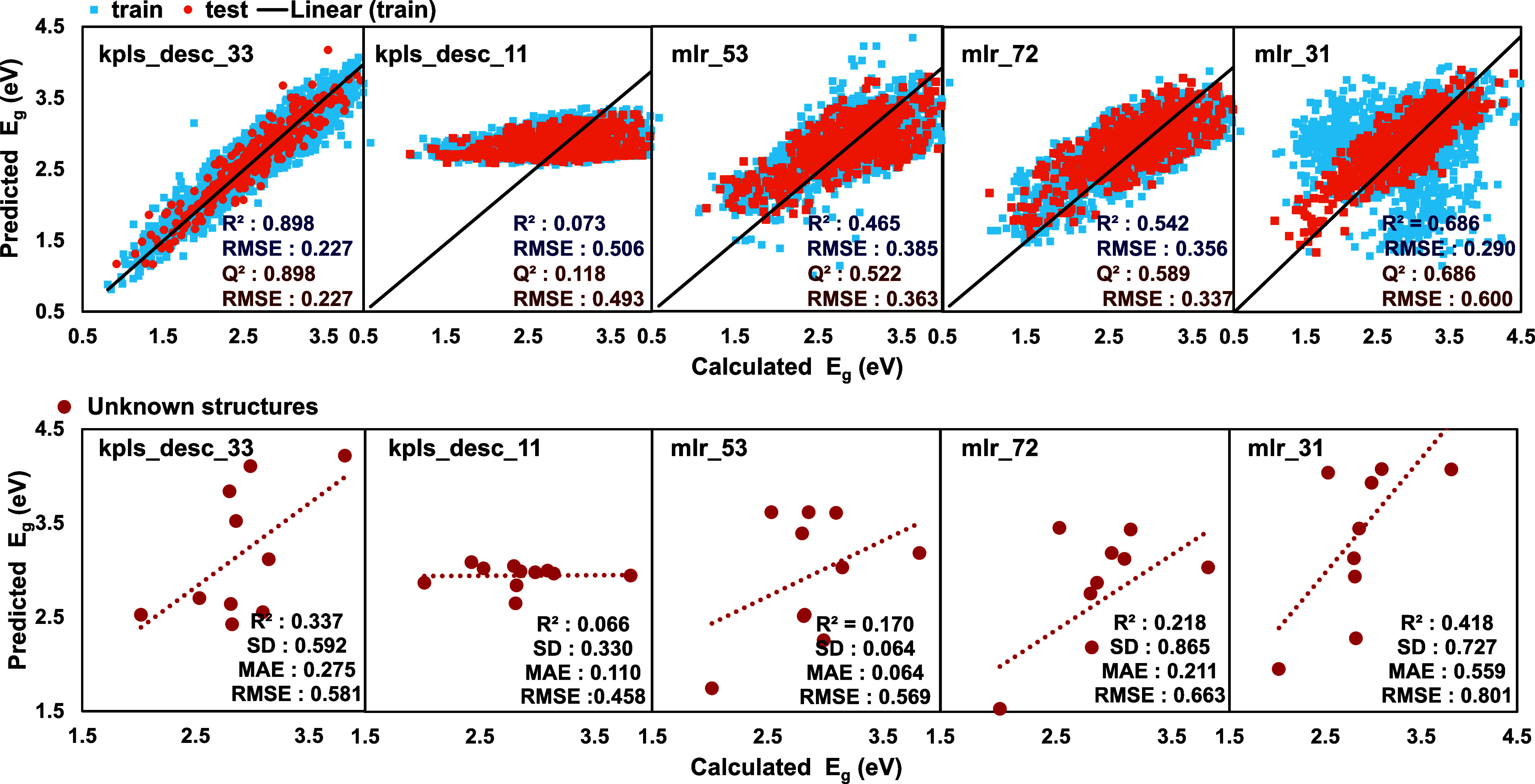
Comparison of predicted and DFT-calculated band gaps for training (blue) and test (orange) sets using **kpls_desc_33**, **kpls_desc_11**, **mlr_53**, **mlr_72**, and **mlr_31**. The black line corresponds to the linear fitting line for the training set. The lower graphs depict predictions for unseen molecules. The red dashed line shows the linear fitting of the prediction for the unknown structures.

Another ML model was constructed using polymer descriptors derived from the molecular structures. For this purpose, the head and tail atoms of D–A monomers are assigned to identify the reactive site where polymerization occurs. Polymer descriptors containing ring atoms fraction, double ring atoms fraction, triple ring atoms fraction, backbone atoms fraction, rotatable bonds fraction, and sp^3^ atoms fraction were calculated for the data set and used for training ML models. Table 1 shows the performance of the constructed ML model using polymer descriptors, **kpls_desc_11.** This method showed a poor performance, achieving an *R* ^2^ of 0.493 and a *Q* ^2^ value of 0.118 as shown in Figure 2. For this method, the optimum number of factors was found to be one, which was not sufficient for the prediction of band gap values. To improve performance, polymer fingerprints were combined with polymer descriptors for the **mlr_53** ML model. This model resulted in a moderate performance with an *R* ^2^ of 0.465 and a *Q* ^2^ value of 0.522. The ML model included 15 independent variables, selected based on their correlation with band gap. While backbone atoms fraction, ring atom fraction, and rotatable bonds fraction made a slight contribution, 17 different polymer fingerprints have stronger influence, both positive and negative impacts on band gap prediction as shown in Figure S4. The coefficient in Table S2 represents the contribution of variables to the band gap prediction; the standard error indicates the variability of the mean estimate and reflects how much the sample mean would vary if the analysis was repeated. and the T value is the representation of the statistical significance of the variable in variation for band gap. Overall, **mlr_53** showed moderate predictive power with relatively low *R* ^2^ and strong polymer fingerprint dependency, meaning that additional descriptors are required for further improvement on band gap prediction.

Next, descriptors based on functional group counts, such as heteroatomic rings, rotatable bonds, aliphatic rings, and aromatic rings, were used in **mlr_72** yielding moderate accuracy with an *R* ^2^ of 0.542 and a *Q* ^2^ value of 0.589, as demonstrated in Figure 2. This model contained 12 independent variables, and the ensemble of the **mlr_72** model is given in Table S3 and Figure S4. Results showed that the number of electron-withdrawing functional groups in the D–A molecules has the highest negative impact on the band gap with a coefficient of −0.00166. Conversely, the number of carbonyl units in D–A molecules has the highest positive contribution, increasing the band gap with a coefficient of 0.00208. Even though the knowledge of variable coefficient is advantageous and the ML model demonstrates a moderate predictive power capturing some key trends, functional group counts do not have sufficient information to enhance the performance of the ML model.

Cheminformatic descriptors including molecular weight, chirality, and polarity were used in **mlr_31**, achieving an *R* ^2^ of 0.686 and a *Q* ^2^ value of 0.686. The variables influencing the model and their respective contributions are detailed in Table S4. Variables such as hydrogen bond donor and acceptor units, number of fluorine atoms, polarity, and sum of carbon atoms in aromatic systems have high T-statistics (above 20), indicating they play a crucial role in determining the prediction as depicted in Figure S4. The lowest contribution belongs to variables such as number of oxygen atoms and average number of methyl groups in the molecule, suggesting they are less influential on the band gap given in the **mlr_31** model.

To evaluate the generalizability of ML models across different fingerprint types, 10 specific D–A-type monomers that are unknown by our algorithms were prepared and examined at the same levels of theory using the B3LYP functional (Figure 3). Only 10 of the possible 36 D–A combinations from the selected donor and acceptor units were included in the unseen test set. These were chosen to cover a representative random chemical space. Although a full combination space could enhance completeness, a focused random subset is opted to maintain computational efficiency and a balanced scope for this study. The band gaps of these unknown test structures cover a broad energy range (1.75–3.75 eV) and include widely used D–A units in the organic electronic applications.^12,37–40^ Although the **kpls_desc_33** resulted in high *R* ^2^ values, the predictive power for the unseen molecules was significantly low, indicating the presence of overfitting where the model performs well on the training data but fails to generalize effectively to unseen test molecules as shown in Figure 2. This suggests that the selected descriptors are data set-specific and do not capture the fundamental relationships governing band gap prediction. The rest of the models (**kpls_desc_11**, **mlr_53**, **mlr_72**, and **mlr_31**) also failed to accurately predict the band gap of unseen test molecules resulting in predictive power lower than 0.418. These models were not successful in training and testing the dataset, even though the data set is considerably large and contains a large number of D–A monomers, suggesting a high redundancy of descriptors, which highlights the importance of feature selection for accurate predictions.

**Figure 3:**
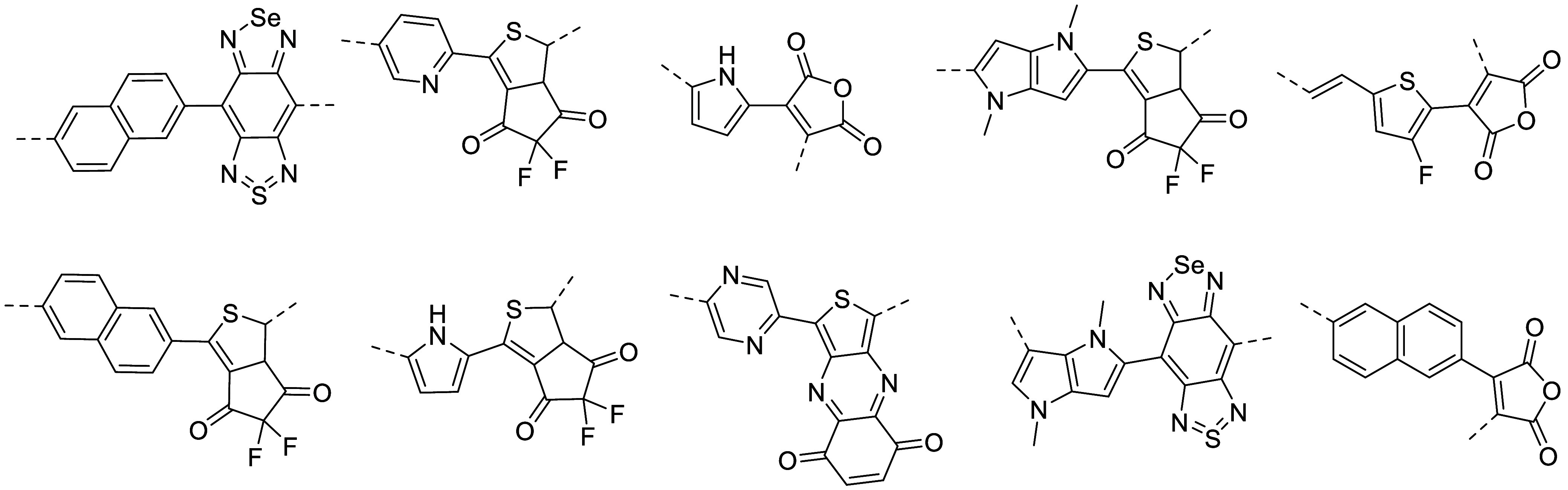
Test set of D–A structures, which are unknown by the ML models.

Four types of binary fingerprints (dendritic, linear, molprint2D, and radial) were generated to characterize the molecular structures and separately employed to analyze their influence on band gap prediction. Results indicated that the KPLS regression model has higher predictive performance compared to other models for binary fingerprints of D–A units as shown in Figure 4. Among the models, **kpls_radial_35**, the radial fingerprints-based model, achieved the highest performance with an *R* ^2^ of 0.899 and a standard deviation of 0.007 for the training set. For the test set, the model achieved an exceptionally high *Q* ^2^ of 0.900 and an RMSE of 0.007, demonstrating its robustness. This ML method relied on radial fingerprints and identified seven optimal factors that influence the band gap prediction. The second-best ML model was molprint2D fingerprints dependent, **kpls_molprint2D_11**, which achieved an *R* ^2^ of 0.895 and a standard deviation of 0.007 for the training set. It demonstrated a *Q* ^2^ of 0.914 and an RMSE of 0.007 for the test set, which is the lowest error in this series indicating consistency in performance across the data set. This is the highest *Q* ^2^ in this comparison, suggesting that the model has the potential to generalize well to unseen data, making this model a strong candidate for real-world applications in material design. Dendritic fingerprints-dependent ML model **kpls_dendritic_26** exhibited lower performance with an *R* ^2^ of 0.855 and an ML model score of 0.826. It demonstrated a *Q* ^2^ of 0.839 and an RMSE of 0.009 for the test set, based on four optimum number of factors, resulting in the lowest model score among the types of binary fingerprints. In addition, linear fingerprints-dependent ML model **kpls_linear_81** resulted in a high *R* ^2^ of 0.891, a *Q* ^2^ of 0.796 and an RMSE of 0.0087 relying on only three optimum factors for the prediction of the band gap.

**Figure 4:**
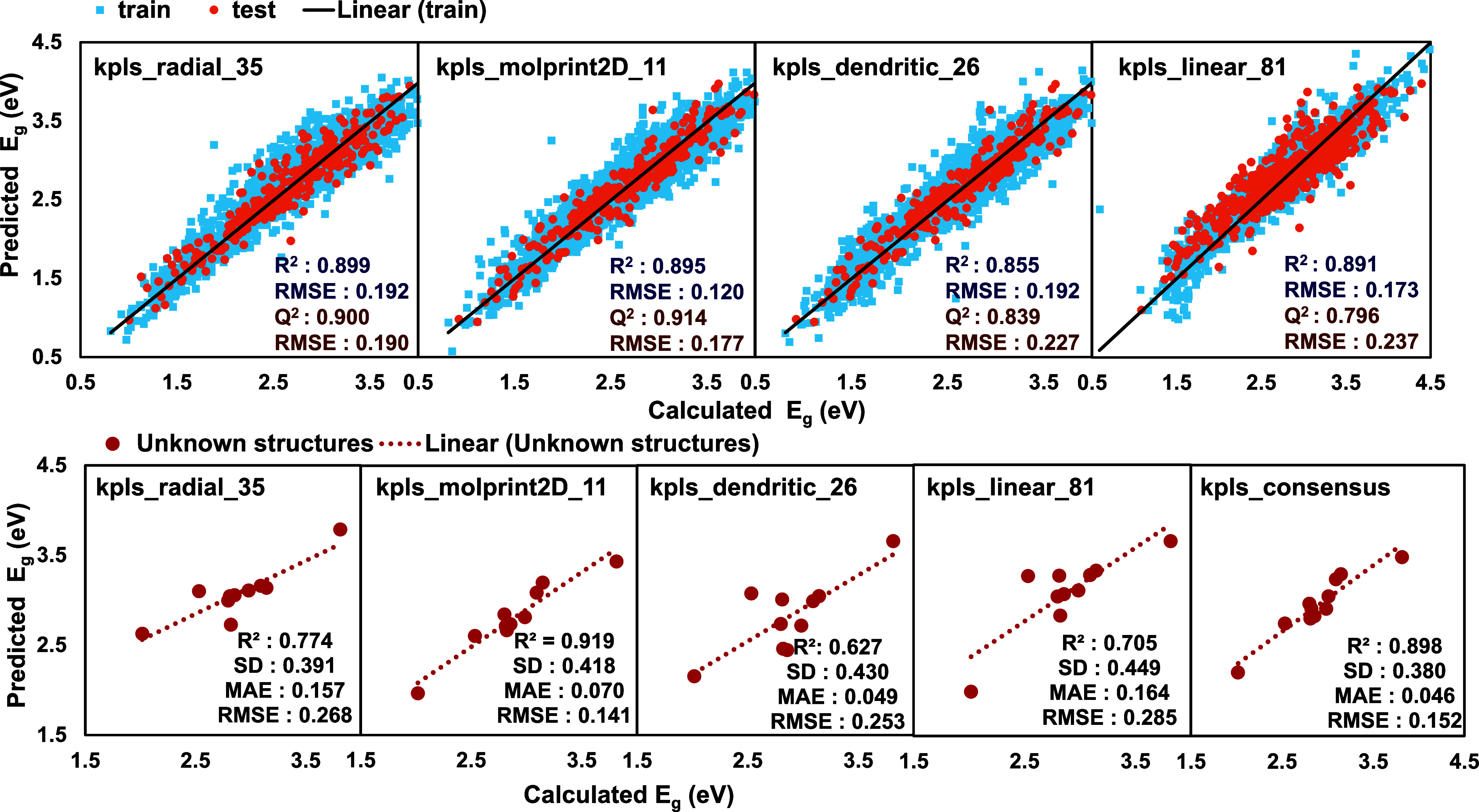
Comparison of predicted and DFT-calculated band gaps for training (blue) and test (orange) sets using **kpls_radial_35**, **kpls_molprint2D_11**, **kpls_dendritic_26**, and **kpls_linear_81**. The black line corresponds to the linear fitting line for the train set. The lower graphs depict predictions for unseen molecules. The lower graphs depict predictions for unseen molecules using **kpls_radial_35**, **kpls_molprint2D_11**, **kpls_dendritic_26, kpls_linear_81**, and **kpls_consensus** where the best 40 models with *R* ^2^ > 0.82 were employed for prediction. The red dashed line shows the linear fitting of prediction for the unknown structures.

The performance of binary fingerprint type-based ML models for the prediction of band gaps for unknown molecules is shown in Figure 4. The predicted band gap for **kpls_molprint2D_11** ML model showed superior performance with an *R* ^2^ of 0.919, a standard deviation of 0.418, and an RMSE of 0.141. Molprint2D fingerprints represent the radial-like fingerprint that captures environments of atoms by encoding lists of atom types positioned at varying topological distances.^41^ In other words, each heavy atom in the structure is defined by an environment that includes all other heavy atoms within a two-bond distance. During the band gap prediction from structural properties, this information gains importance since it enables the ML models to better understand the local atomic interactions and their influence on electronic properties. Compared with other fingerprint types, such as dendritic which counts the linear and branched fragments in the molecule or linear fingerprints which are also referred to as extended connectivity fingerprints, the Molprint2D fingerprints provide a more comprehensive representation of molecular topology.^42,43^ The ability to incorporate detailed topological features contributes to the reduction of prediction errors, resulting in the lowest RMSE in this comparison (RMSE of 0.152). To improve generalizability, we constructed a consensus model averaging predictions from top-performing models (*R* ^2^ > 0.82), yielding robust performance for unseen test cases. This ML model, **kpls_consensus** where the top 40 ML models with R2 > 0.820 were combined and taken an average value for the predicted band gap, resulting in an R2 of 0.898. The **kpls_radial_35** ML model, which performed best in model construction, showed a significant drop in prediction performance for unseen molecules (an *R* ^2^ of 0.774). The reason for this is that radial fingerprints define molecular structures based on circular atom neighborhoods which did not capture relevant features for predicting the band gap of unseen molecules.^44^ The **kpls_dendritic_26** ML model which encodes both linear and branched fragment properties as well as linear paths with a maximum of five bonds per path exhibited a moderate success with an *R* ^2^ of 0.627 due to the insufficient ability to capture ring structures, which are prevalent in the unseen test set. Similarly, the **kpls_linear_81** ML model also demonstrated moderate predictive performance because linear-type fingerprints capture only linear paths and fail to account for the more complex, nonlinear environments of atoms within the molecule, which are critical for accurately predicting the band gap. It is also possible to combine numerical descriptors and binary fingerprints in a single ML model. However, KPLS models that integrate fingerprints with descriptors have shown, on average, no improvement in predictive performance for the target property compared with models that rely only on fingerprints.^36^ The combined analysis of numerical descriptors and binary fingerprints resulted in the best performance with the **kpls_desc_radial** ML model achieving an *R* ^2^ of 0.774, matching the performance of **kpls_radial_35**. This indicates that numerical descriptors have no significant impact on band gap prediction for unseen molecules in the KPLS method.

### QSPR on Reorganization Energy Prediction

According to the semiclassical Marcus equation, the pre-exponential factor for the hole hopping rates between two adjacent materials is inversely proportional to λ_h_, which is associated with the charge transport.^16^ Given this relationship, accurately predicting λ_h_ requires selecting descriptors that effectively capture the key molecular and electronic factors influencing the charge transport. As in the previous section, different descriptor types were separately assigned to the ML models to determine the most effective type for predicting λ_h_, using the same train-to-test split. The results are summarized in Table 2 and visualized in Figure 5. Analysis based on 2D-binary fingerprints resulted in the **kpls_radial_27** ML model achieving an *R* ^2^ of 0.748 and an RMSE of 0.077 for the training set. For the test set, the model achieved a *Q* ^2^ of 0.753 and an RMSE of 0.771, with seven optimal factors contributing to the prediction. The second ML model, based on numerical descriptors, performed similarly, with **kpls_desc_39** achieving an *R* ^2^ of 0.751 and an RMSE of 0.077 for the training set and a *Q* ^2^ of 0.747 and an RMSE of 0.772 for the test set, relying on 23 optimal factors. When binary fingerprints were combined with numerical descriptors, the best-performing model remained as **kpls_radial_27**, indicating the advantages of binary fingerprints over numerical descriptors.

**Figure 5:**
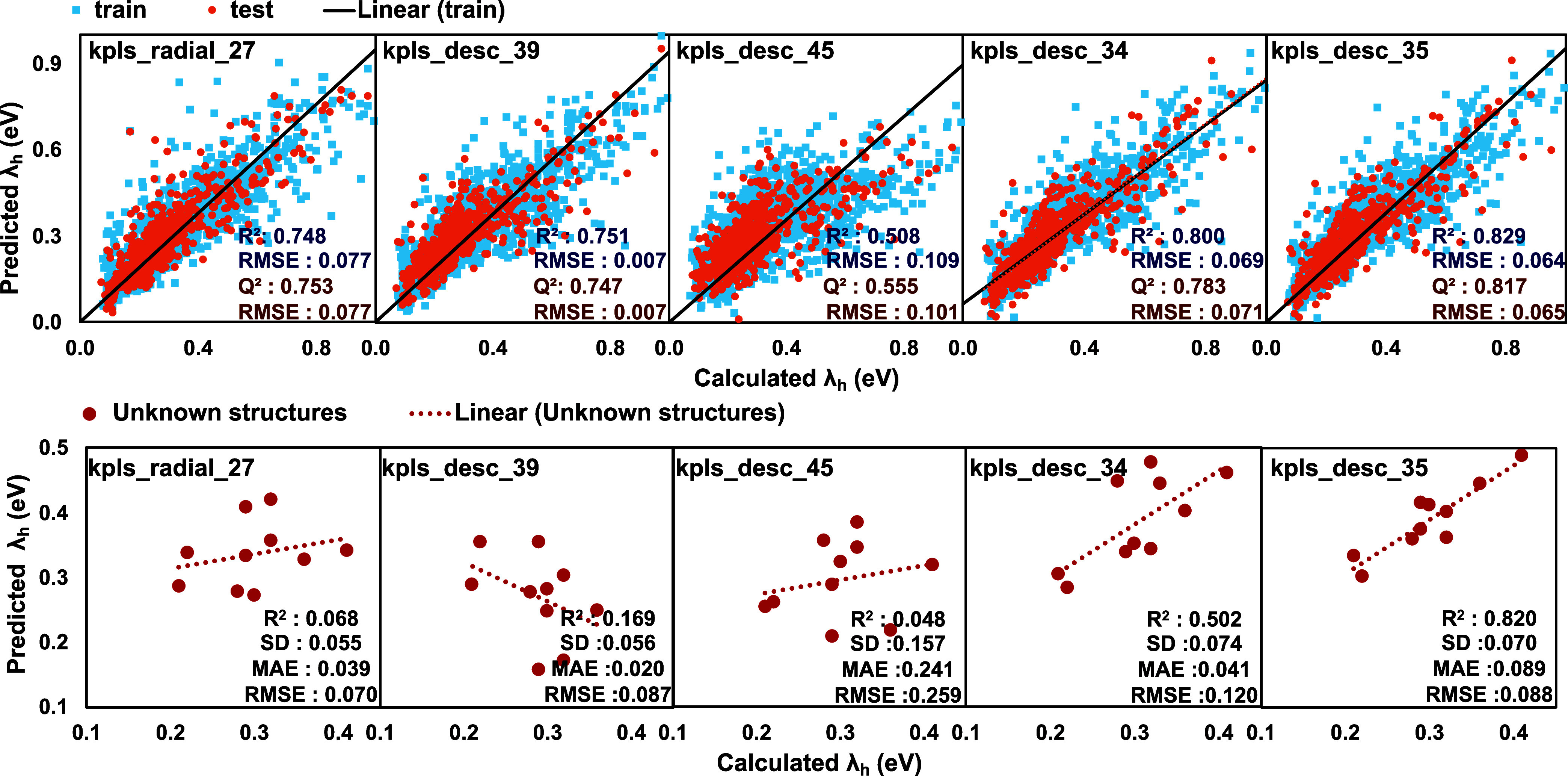
Comparison of predicted and DFT-calculated hole reorganization energy for training (blue) and test (orange) sets using **kpls_radial_27**, **kpls_desc_39**, **kpls_desc_45**, **kpls_desc_34**, and **kpls_desc_35**. The black line corresponds to the linear fitting line for the training set. The lower graphs depict a prediction for unknown molecules. The red dashed line shows the linear fitting of prediction for the unknown structures.

**Table 2:** Performance of ML Models Trained and Tested for Reorganization Energy

**ML model**	**model score**	**RMSE**_**train**_	***R*** ^**2**^	**RMSE**_**test**_	***Q*** ^**2**^
**kpls_radial_27**	0.748	0.077	0.748	0.771	0.753
**kpls_desc_39**	0.748	0.077	0.751	0.772	0.747
**kpls_desc_4**	0.245	0.142	0.158	0.131	0.236
**mlr_70**	0.443	0.121	0.383	0.109	0.505
**kpls_desc_61**	0.419	0.121	0.355	0.115	0.422
**kpls_desc_45**	0.535	0.109	0.508	0.101	0.554
**kpls_desc_34**	0.775	0.069	0.800	0.071	0.783
**kpls_desc_35**	0.812	0.064	0.829	0.066	0.817

In contrast, polymer descriptor-based analysis failed to train an effective model. The best polymer descriptor-dependent model, **kpls_desc_4**, achieved an *R* ^2^ of only 0.158 with an RMSE of 0.142 for the training set and a *Q* ^2^ of 0.236 and an RMSE of 0.131 for the test set, as illustrated in Figure S5. These results demonstrate the inability of polymer descriptors to predict λ_h_, unlike band gap energy which strongly depends on electron delocalization and conjugation and can be effectively described by topological descriptors. Reorganization energy is a dynamic property influenced by structural relaxation, bond length changes, and nuclear reorganization where none of these factors can be captured by static structure-based descriptors.

When polymer descriptors were combined with the polymer fingerprints, the best-performing model, **mlr_70**, achieved an *R* ^2^ of 0.383 with an RMSE of 0.121 for the training set and a *Q* ^2^ of 0.505 with an RMSE of 0.109 (Figure S5). This suggests that incorporating polymer descriptors alongside polymer fingerprints enhances prediction compared to that with polymer descriptors alone (**kpls_desc_4)**. However, high RMSE indicates data set inhomogeneity, making it challenging for the ML model to fit all of the data effectively. The diverse D–A units included in our data set contribute to this variation and the high RMSE suggests that the training and test distributions do not fully overlap, leading to poor test performance. Figure S6 highlights the 20 most influential variables in **mlr_70**, revealing that the highest contributions come from D–A unit-dependent fingerprints rather than generalized polymer features.

Another approach involved training models based on counts of specific functional groups in the D–A molecules. This yielded the **kpls_desc_61** model with an *R* ^2^ of 0.355 for the training set and a *Q* ^2^ value of 0.422 for the test set, with an RMSE of 0.115. A moderate performance demonstrates that functional group counts are insufficient for accurately predicting hole reorganization energy, as demonstrated in Figure S5. The cheminformatic descriptor-based ML model, **kpls_desc_45**, performed slightly better, achieving an *R* ^2^ of 0.508 for the training set and a *Q* ^2^ of 0.554 for the test set, using 12 optimal factors. However, RMSE values for the training and test sets (0.109 and 0.101, respectively) were high, suggesting that cheminformatic descriptors and functional group counts fail to capture charge transfer behavior or establish a meaningful correlation with reorganization energy. As these RMSE values are lower than those for other models, they are still considered high relative to the expected accuracy for predicting reorganization energy. While functional groups influence electronic properties, their contribution to the reorganization energy prediction is minimal.

The performance of ML models was also evaluated using unseen molecules, as shown in Figure 5. Although model score values for **kpls_radial_27** and **kpls_desc_39** exceeded 0.70, their predictions of hole reorganization energy in unseen molecules were unsuccessful, yielding *R* ^2^ values of 0.068 and 0.169, respectively. Additional methods (**kpls_desc_4**, **mlr_70, kpls_desc_61**, and **kpls_desc_45**) also demonstrated weak generalization performance with *R* ^2^ values of 0.150, 0.204, 0.206, and 0.048, respectively. These results indicate a common limitation among the tested models: the selected descriptors did not adequately represent the fundamental physicochemical and electronic factors that govern reorganization energy, even though they proved effective in predicting band gap.

To address these limitations, the best-performed model (**kpls_desc_39**) was retested with the band gap energy included as an additional descriptor during training and testing. Figure 5 shows the improved performance for the **kpls_desc_34** ML model, which was generated with band gap energy information. This suggests that incorporating band gap values as descriptors offers limited compensation for the shortcomings of the original descriptor sets. Band gap is an electronic property that reflects the energy difference between the highest occupied and lowest unoccupied molecular orbitals. While it has relevance to charge transport, it does not directly account for the specific structural reorganization processes involved in hole reorganization energy. As a result, the prediction performance for hole reorganization energy on unseen molecules improved to a moderate level.

To further improve the performance, frontier molecular orbital energy levels, which were calculated by DFT calculations, were combined with numerical descriptors; the performance of the **kpls_desc_35** ML model resulted in an *R* ^2^ of 0.829 and *Q* ^2^ of 0.817 which represents one of the best-performing models for the prediction of hole reorganization energy. Since reorganization energy is affected by the changes in molecular geometry and electron distribution between the neutral and charged states, especially the HOMO and LUMO energy levels play a dominant role in its prediction. Molecules with rigid, planar structures and delocalized orbitals undergo minimal geometric relaxation, resulting in lower reorganization energy. In contrast, flexible structures with localized orbitals tend to experience greater relaxation and therefore higher reorganization energy. While band gap prediction performed best with binary fingerprints, reorganization energy prediction was improved when by integrating improved when quantum descriptors such as HOMO–LUMO energy levels were included -since reorganization energy reflects intramolecular relaxation dynamics, requiring deeper electronic structure information. **kpls_desc_34** and **kpls_desc_35** showed successful predictive power for hole reorganization energy, achieving an *R* ^2^ of 0.820 for unseen test molecules, demonstrating the potential of HOMO–LUMO energy levels as key features for improving model accuracy. Since the HOMO and LUMO energies provide insight into molecular polarization, charge localization, and the relative stability of charged species, they are critical for understanding how a molecule responds to gaining or losing an electron. By incorporating this information into the ML model, it becomes possible to capture both the electronic and the geometric aspects that are essential for accurate calculation of reorganization energy.

## Conclusions

We developed and evaluated ML-driven models for predicting band gap and charge carrier reorganization energy in donor–acceptor conjugated polymers, which is a key class of materials for organic photovoltaics. By curating a large data set of 3120 CPs consisting of 60 donor and 52 acceptor units, we ensured a diverse and representative selection of materials, laying the groundwork for accurate and generalizable ML predictions. Our analysis demonstrated that the choice of molecular descriptors and fingerprints significantly impacts the ML model performance. KPLS regression with radial and molprint2D fingerprints provided the highest accuracy for band gap prediction, while frontier orbital energy levels played a crucial role in improving the reorganization energy predictions. In contrast, polymer descriptors alone showed limited predictive capability, highlighting the importance of selecting the most relevant structural and electronic descriptors for reliable property predictions. Beyond performance evaluation, this study provided insights into why certain descriptors show a higher success rate, especially for reorganization energy predictions, which remain challenging due to the dynamic nature of charge mobility. Combinatorial quantum mechanical and ML techniques can significantly improve predictive accuracy, providing a cost-effective alternative to traditional experimental and computational approaches for organic electronics. Beyond raw predictive accuracy, our results emphasize the physical relevance and model interpretability of different descriptor types, illustrating how the molecular structure and electronic property interplay vary across prediction targets. The insights presented here contribute to a more informed and systematic approach to feature selection in ML-guided materials design, while the accompanying data set serves as a valuable benchmark for future developments in the field.

## Supplementary Material







## Notes

Donor and acceptor units used to construct the D-A data set, data set construction scheme, details of ML models for the prediction of band gap and hole reorganization energy, variables and contribution coefficient for ML models, and predictive power graphs of additional ML models for reorganization energy prediction (PDF)
Optimized structures of 3120 D–A (ZIP)
Reports for ML models, calculated and predicted values of target properties, list of all descriptors, fingerprints, band gaps, and hole and electron reorganization energies for the data set (ZIP)
