# Supplementary material for: Band Gap and Reorganization Energy Prediction of Conducting Polymers by the Integration of Machine Learning and Density Functional Theory
Source: J Chem Inf Model. 2025 May 28;65(11):5360–9. doi: 10.1021/acs.jcim.5c00345 (PMC12152970; doi:10.1021/acs.jcim.5c00345)
Supplement: Supplementary file 1 [file ci5c00345_si_001.pdf]

Band Gap and Reorganization Energy Prediction of Conducting Polymers by the Integration of Machine Learning and Density Functional Theory

Tugba Haciefendioglu [α], *Erol Yildirim** [α, β, γ]

[α] Department of Chemistry, Middle East Technical University, 06800 Ankara, Turkey

[β] Department of Polymer Science and Technology, Middle East Technical University,06800 Ankara, Turkey

[γ] Department of Micro- and Nanotechnology, Middle East Technical University, 06800 Ankara, Turkey

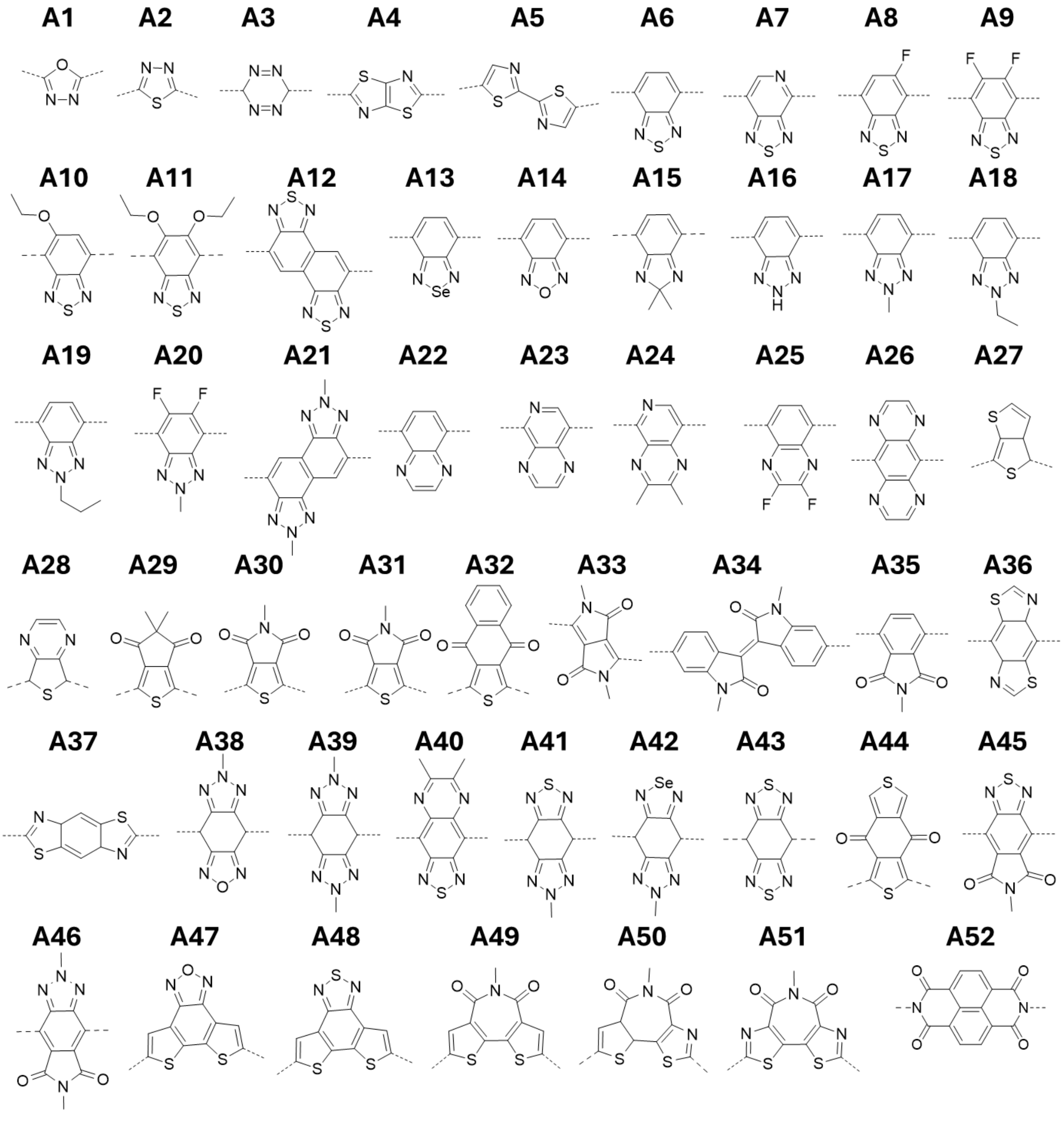

Figure S1. 52 acceptor units conducted for dataset construction.

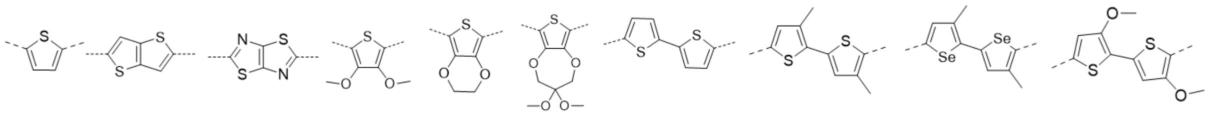

D1 D2 D3 D4 D5 D6 D7 D8 D9 D10

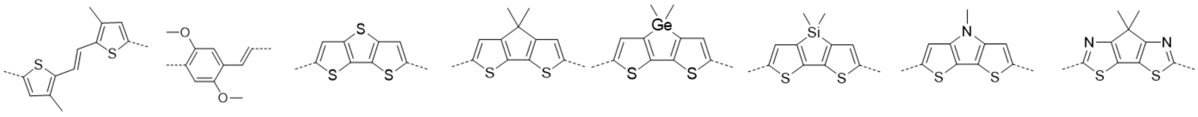

D11 D12 D13 D14 D15 D16 D17 D18

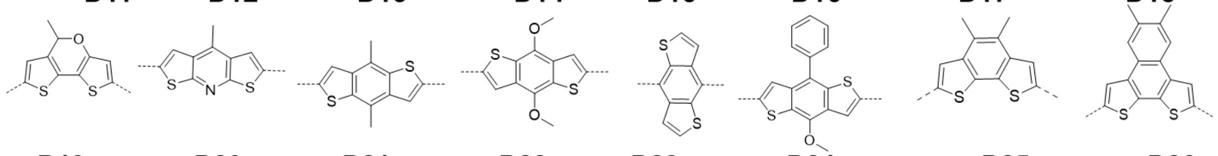

D19 D20 D21 D22 D23 D24 D25 D26

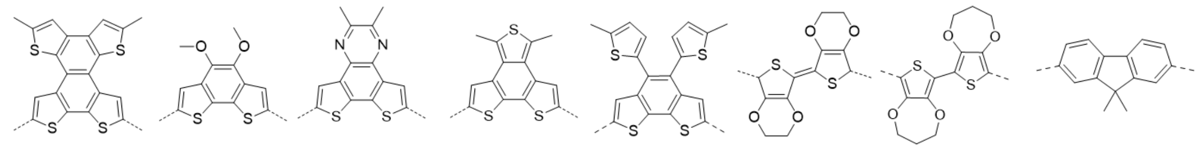

D27 D28 D29 D30 D31 D32 D33 D34

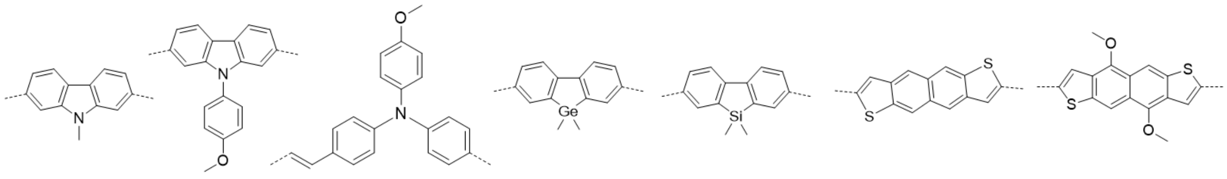

D35 D36 D37 D38 D39 D40 D41

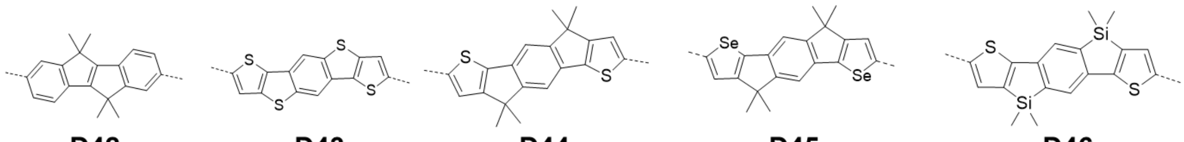

D42 D43 D44 D45 D46

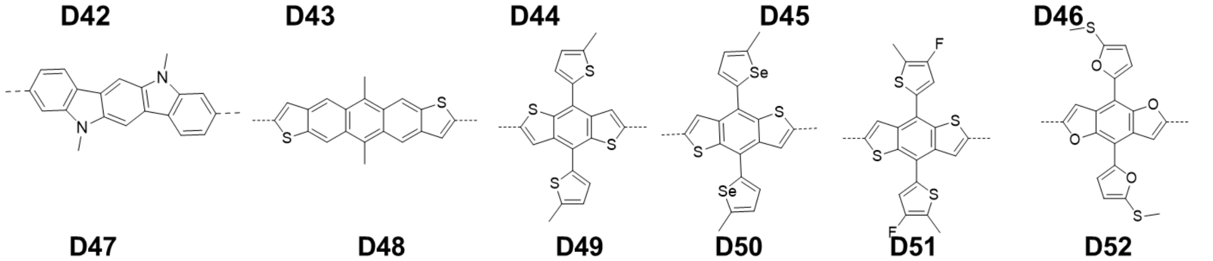

D47 D48 D49 D50 D51 D52

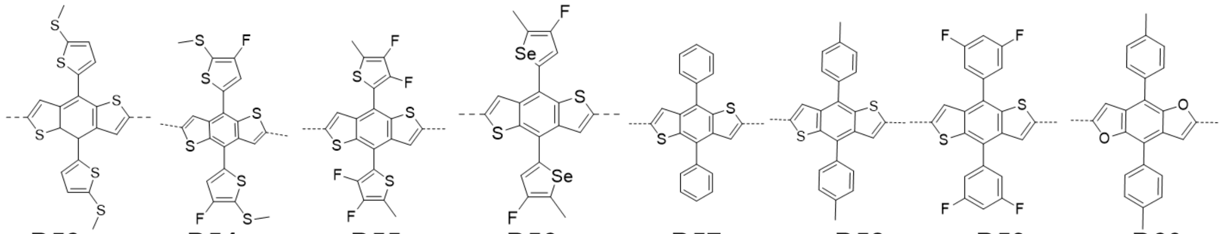

D53 D54 D55 D56 D57 D58 D59 D60

Figure S2. 60 donor units conducted for dataset construction.

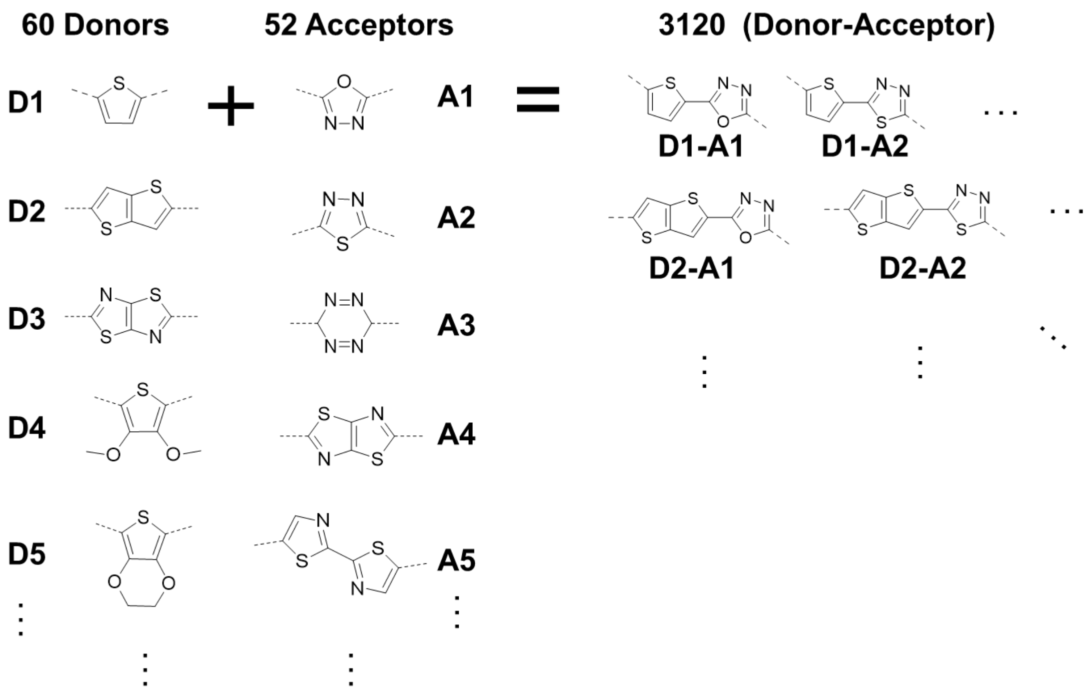

Figure S3. Dataset construction using donor-acceptor units.

Table S1. Details of ML models for the predicition of band gap and hole reorganization energy (for futher details see Supporting Information (rar)).

ML Model	Method	Feature Selection
**kpls_desc_33**	KPLS	Numerical descriptors
**kpls_desc_11**	KPLS	Polymer descriptors
**mlr_53**	MLR	Polymer descriptors + Polymer fingerprints
**mlr_72**	MLR	Functional group counts
**mlr_31**	MLR	Cheminformatic descriptors
**kpls_radial_35**	KPLS	Binary fingerprints (radial)
**kpls_molprint2D_11**	KPLS	Binary fingerprints (molprint2D)

**kpls_dendritic_26**	KPLS	Binary fingerprints (dendritic)
**kpls_linear_81**	KPLS	Binary fingerprints (linear)
**kpls_consesnsus**	KPLS	Top 40 ML models with R^2> 0.820 (consensus prediction)
**kpls_desc_radial**	KPLS	Numerical descriptors + Binary fingerprints
**kpls_radial_27**	KPLS	2D-binary fingerprints
**kpls_desc_39**	KPLS	Numerical descriptors
**kpls_desc_4**	KPLS	Polymer descriptors
**mlr_70**	MLR	Polymer descriptors + Polymer fingerprints
**kpls_desc_61**	KPLS	Functional group counts
**kpls_desc_45**	KPLS	Cheminformatic descriptors
**kpls_desc_34**	KPLS	Numerical descriptors + Band gap energy
**kpls_desc_35**	KPLS	Numerical descriptors + Frontier orbital energy

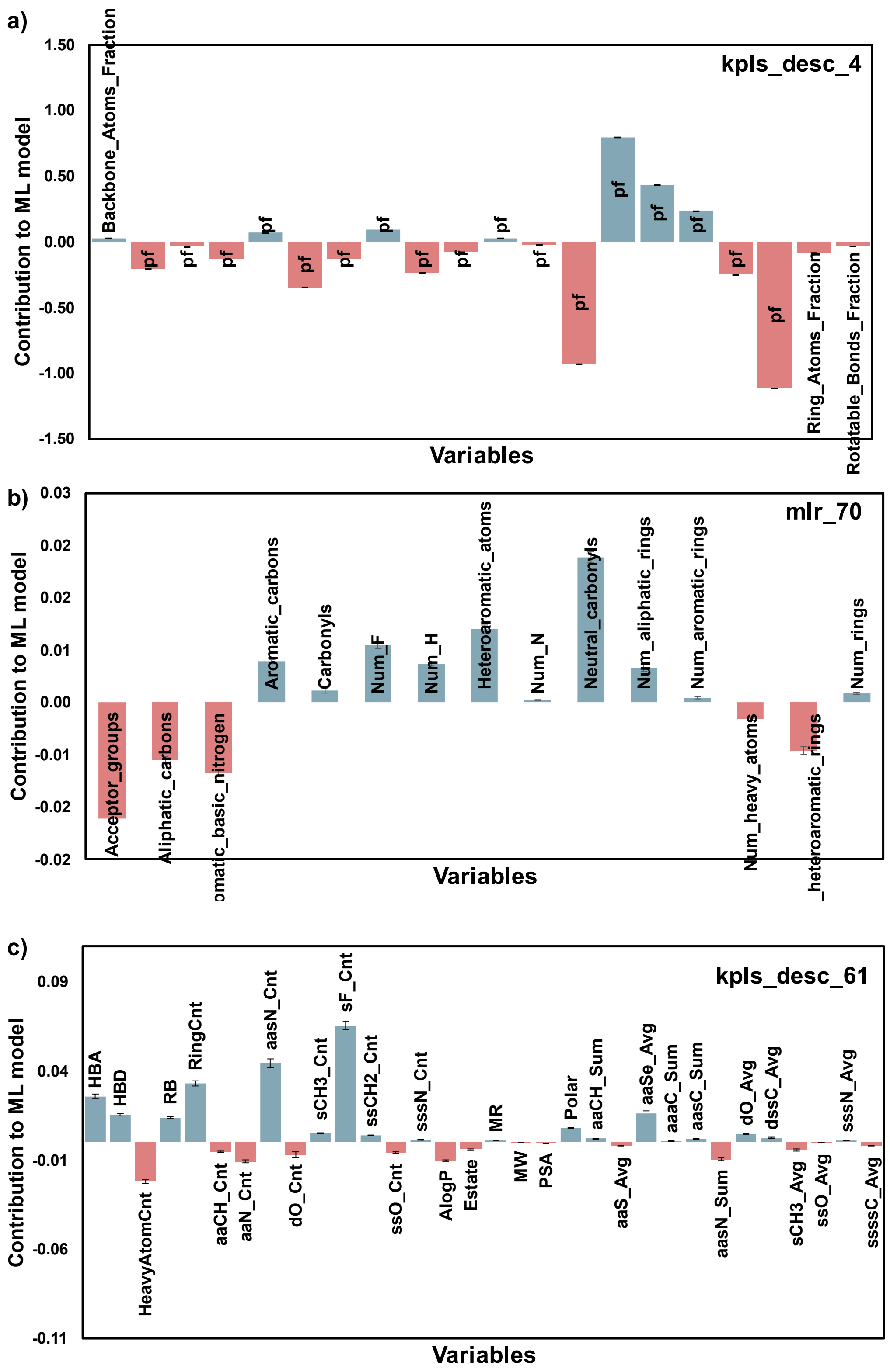

Figure S4. Variables and contribution coefficient of these variables to the a) **kpls_desc_4**, b) **mlr_70** and c) **kpls_desc_61** models. Blue and red bars represent the increasing and decreasing variables for band gap, respectively.

Table S2. Ensemble **mlr_53** ML model summary.*

**Coefficient**	**Standard Error**	**T value**	**Variable**
$3.05x10^{-2}$	$2.88x10^{-3}$	10.5779	Backbone_Atoms_Fraction
$-2.04x10^{-1}$	$2.36x10^{-2}$	8.6332	pf
$-3.42x10^{-2}$	$3.42x10^{-3}$	10.0041	pf
$-1.28x10^{-1}$	$2.12x10^{-2}$	6.045	pf
$7.17x10^{-2}$	$6.54x10^{-3}$	10.9556	pf
$-3.43x10^{-1}$	$2.81x10^{-2}$	12.176	pf
$-1.30x10^{-1}$	$1.20x10^{-2}$	10.7982	pf
$9.66x10^{-2}$	$7.59x10^{-3}$	12.7306	pf
$-2.32x10^{-1}$	$1.07x10^{-2}$	21.6082	pf
$-7.26x10^{-2}$	$9.89x10^{-3}$	7.3433	pf
$3.02x10^{-2}$	$3.75x10^{-3}$	8.0476	pf
$-2.03x10^{-2}$	$5.09x10^{-3}$	3.988	pf
$-9.24x10^{-1}$	$3.96x10^{-2}$	23.3548	pf
$8.00x10^{-1}$	$1.08x10^{-1}$	7.4235	pf
$4.36x10^{-1}$	$3.35x10^{-2}$	12.9974	pf
$0.238x10^{-1}$	$1.87x10^{-2}$	12.7461	pf
$-2.47x10^{-1}$	$8.33x10^{-3}$	29.6122	pf
-1.11	$1.26x10^{-1}$	8.811	pf
$-8.02x10^{-1}$	$5.82x10^{-3}$	13.7733	Ring_Atoms_Fraction
$-2.74x10^{-2}$	$4.42x10^{-3}$	6.2112	Rotatable_Bonds_Fraction

*pf: polymer fingerprints

Table S3. Ensemble **mlr_72** ML model summary.

Coefficient	Standard Error	T value	Variable
$-1.66x10^{-2}$	$6.93x10^{-4}$	23.99	Acceptor_groups
$-8.29x10^{-3}$	$5.08x10^{-4}$	16.31	Aliphatic_carbons
$-1.01x10^{-2}$	$5.19x10^{-4}$	19.50	Aromatic_basic_nitrogens
$5.89x10^{-3}$	$3.25x10^{-4}$	18.12	Aromatic_carbons
$1.69x10^{-3}$	$3.08x10^{-4}$	5.48	Carbonyls
$8.24x10^{-3}$	$4.68x10^{-4}$	17.62	Number_of_F_atoms
$5.51x10^{-3}$	$2.39x10^{-4}$	23.07	Number_of_H_atoms
$1.05x10^{-2}$	$6.13x10^{-4}$	17.16	Heteroaromatic_atoms
$3.73x10^{-4}$	$7.73x10^{-5}$	4.82	Number_of_N_atoms
$2.08x10^{-2}$	$7.97x10^{-4}$	26.08	Neutral_carbonyls
$4.98x10^{-3}$	$5.18x10^{-4}$	9.61	Number_aliphatic_rings
$7.05x10^{-4}$	$1.70x10^{-4}$	4.14	Number_aromatic_rings
$-2.33x10^{-3}$	$2.41x10^{-4}$	9.69	Number_heavy_atoms
$-6.85x10^{-3}$	$5.89x10^{-4}$	11.62	Number_heteroaromatic_rings
$1.32x10^{-3}$	$1.31x10^{-4}$	10.11	Number_of_rings
$4.05x10^{-4}$	$9.31x10^{-5}$	4.35	Tertiary_amines_or_amides

Table S4. Ensemble **mlr_31** ML model summary.*

Coefficient	Standard Error	T value	Variable
$2.59x10^{-2}$	$1.18x10^{-3}$	22.00	HBA

$1.54x10^{-2}$	$6.12x10^{-4}$	25.13	HBD
$-2.19x10^{-2}$	$1.04x10^{-3}$	21.12	Heavy_Atom_Cnt
$1.39x10^{-2}$	$5.63x10^{-4}$	24.75	RB
$3.29x10^{-2}$	$1.40x10^{-3}$	23.56	RingCnt
$-5.50x10^{-3}$	$3.95x10^{-4}$	13.93	aaCH_Cnt
$-1.07x10^{-2}$	$7.06x10^{-4}$	15.20	aaN_Cnt
$4.44x10^{-2}$	$2.36x10^{-3}$	18.80	aasN_Cnt
$-7.01x10^{-3}$	$1.80x10^{-3}$	3.90	dO_Cnt
$5.05x10^{-3}$	$2.07x10^{-4}$	24.35	sCH3_Cnt
$6.57x10^{-2}$	$2.25x10^{-3}$	29.15	sF_Cnt
$3.89x10^{-3}$	$1.72x10^{-4}$	22.66	ssCH2_Cnt
$-5.90x10^{-3}$	$4.38x10^{-4}$	13.47	ssO_Cnt
$1.32x10^{-3}$	$1.81x10^{-4}$	7.28	sssN_Cnt
$-1.04x10^{-2}$	$4.31x10^{-4}$	24.25	AlogP
$-4.06x10^{-3}$	$3.42x10^{-4}$	11.87	Estate
$1.09x10^{-3}$	$1.08x10^{-4}$	10.08	MR
$-6.81x10^{-5}$	$8.44 x10^{-6}$	8.06	MW
$-3.61x10^{-4}$	$2.26x10^{-5}$	15.97	PSA
$8.03x10^{-3}$	$2.95x10^{-4}$	27.24	Polar
$2.08x10^{-3}$	$1.44x10^{-4}$	14.37	aaCH_Sum
$-1.96x10^{-3}$	$2.32x10^{-4}$	8.43	aaS_Avg
$1.63x10^{-2}$	$1.39x10^{-3}$	11.71	aaSe_Avg
$8.09x10^{-4}$	$6.67x10^{-5}$	12.14	aaaC_Sum
$2.01x10^{-3}$	$1.35x10^{-4}$	14.94	aasC_Sum

-9.50×10^{-3}	7.99×10^{-4}	11.88	aasN_Sum
4.71×10^{-3}	1.72×10^{-4}	27.43	dO_Avg
2.45×10^{-3}	3.08×10^{-4}	7.95	dssC_Avg
-4.39×10^{-3}	6.43×10^{-4}	6.83	sCH3_Avg
-2.27×10^{-4}	2.19×10^{-5}	10.36	ssO_Avg
1.12×10^{-3}	1.48×10^{-4}	7.56	sssN_Avg
-1.81×10^{-3}	1.91×10^{-4}	9.48	ssssC_Avg

*HBA: Hydrogen Bond Acceptor, HBD: Hydrogen Bond Donor, HeavyAtomCount: The count of atoms other than hydrogen, RB: The number of rotatable bonds in the molecule, RingCount: The count of rings, aaCH_Counnt: Count of aromatic carbon-hydrogen (C-H) bonds in aromatic systems, aaN_Cnt: Count of aromatic nitrogen (N) atoms in aromatic systems, aasN_Cnt: Count of nitrogen atoms in aromatic systems, likely a measure of nitrogen-substituted aromatics, dO_Cnt: Count of donor oxygen atoms in the molecule, often associated with hydrogen bonding, sCH3_Cnt: Count of methyl groups ($-CH_3$) attached to the structure, sF_Cnt: Count of fluorine atoms in the molecular structure, ssCH2_Cnt: Count of methylene (-CH-) groups, ssO_Cnt: Count of oxygen atoms, sssN_Cnt: Count of nitrogen atoms that are part of secondary or tertiary nitrogen structures, AlogP: a measure of hydrophobicity, Estate: The electronic state of the molecule, MR: Molecular Refractivity, a measure of the size and polarizability of the molecule, MW: Molecular Weight, PSA: Polar Surface Area, Polar: A descriptor related to the polar nature of the molecule, possibly indicating its ability to form dipole interactions, aaCH_Sum: Sum of aromatic carbon-hydrogen bonds in the molecule, aaS_Avg: Average sulfur content in aromatic systems, aaSe_Avg: Average selenium content in aromatic systems, aaaC_Sum: Sum of aromatic carbon atoms, aasC_Sum: Sum of carbon atoms in aromatic systems with specific substitutions, aasN_Sum: Sum of nitrogen atoms in aromatic systems

with substitutions, dO_Avg: Average number of donor oxygen atoms in the molecule, dssC_Avg: Average number of carbon atoms involved in double bonds (C=C or C=O) in the molecule, sCH3_Avg: Average number of methyl (-CH_3) groups in the molecule, ssO_Avg: Average number of oxygen atoms in the molecule, sssN_Avg: Average number of nitrogen atoms, ssssC_Avg: Average number of carbon atoms in the molecule involved in substituted aromatic systems.

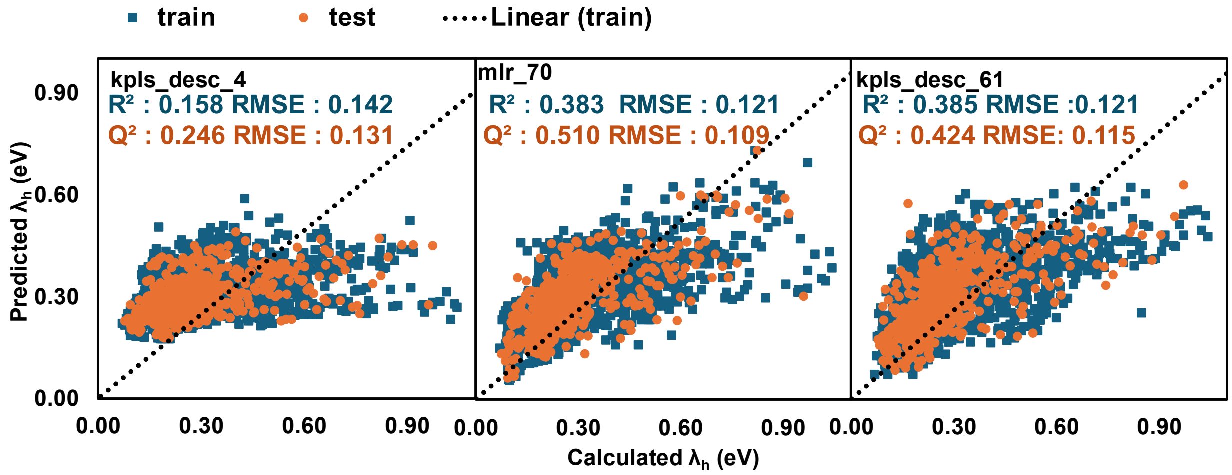

Figure S5. Comparison of predicted and DFT-calculated hole reorganization energy for training (blue) and test (orange) sets using **kpls_desc_4**, **mlr_70** and **kpls_desc_61**. The black line corresponds to linear fitting line for the training set.

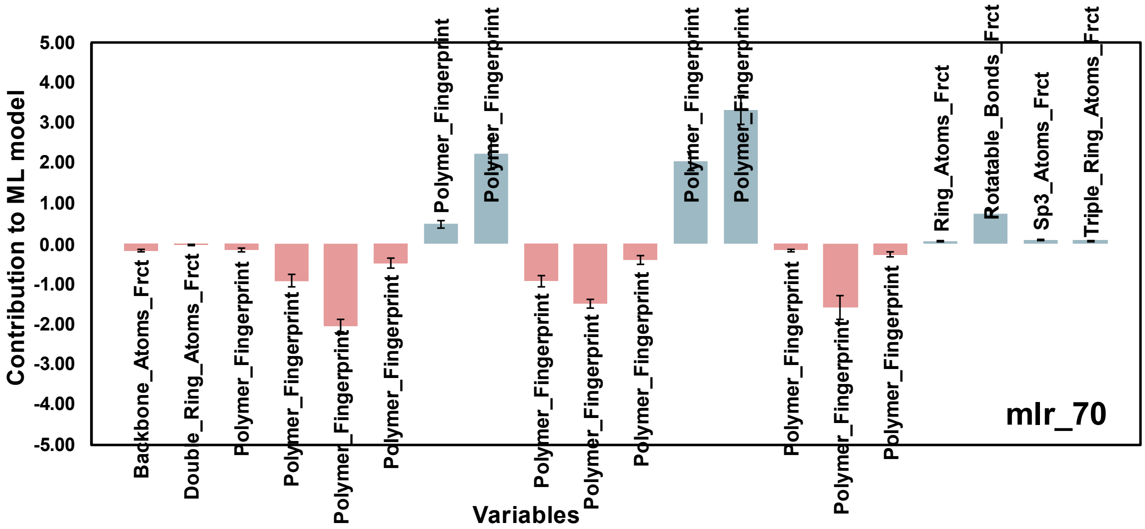

Figure S6. Variables and contribution coefficient of these variables to the **mlr_70.** Blue and red bars represent the increasing and decreasing variables for hole reorganization energy, respectively.
